# Influence of Recharging Wells, Sanitary Collectors and Rain Drainage on Increase Temperature in Pumping Wells on the Groundwater Heat Pump System

**DOI:** 10.3390/s21217175

**Published:** 2021-10-28

**Authors:** Stjepan Strelec, Kristijan Grabar, Jasmin Jug, Nikola Kranjčić

**Affiliations:** 1 Faculty of Geotechnical Engineering, University of Zagreb, Hallerova aleja 7, 42000 Varaždin, Croatia; stjepan.strelec@gfv.unizg.hr (S.S.); nikola.kranjcic@gfv.unizg.hr (N.K.); 2 SPP d.o.o., Koprivnička ulica 47, 42000 Varaždin, Croatia; kristijan@spp.hr

**Keywords:** pumping test, aquifer parameters, sensors, pumping and recharging well, isothermal groundwater maps, influence radius, temperature breakthrough

## Abstract

The utilization of groundwater is becoming increasingly popular for heating and cooling buildings, as well as to regulate the temperature needs of industrial processes. Groundwater has excellent energy potential from various factors, of which environmental acceptability stands out, as groundwater is considered a source of renewable energy. Due to the water table depth below the surface, atmospheric conditions have a negligible effect on the temperature of groundwater, resulting only in minor annual temperature variations, thus also making groundwater a source of reliable renewable energy. This paper presents some aspects of the groundwater heat pump (GWHP) system’s design and addresses a particular problem on the influence of recharge temperature field as well as local utility lines on the pumping well water temperature. An example is given of a system designed for a production hall in the northern part of Croatia. Geological and hydrogeological conditions at the site are highly favourable regarding the groundwater temperature and aquifer parameters. For the needs of this research, precise electronic sensors with data loggers were installed inside the wells. Probe type GSR 120 NT manufactured by Eltratec, Slovenia, is capable of monitoring level, temperature, and electrical conductivity, including telemetric data transfer to the remote server. Mapping the obtained data revealed significant temperature breakthroughs from the recharge wells, as well as local temperature field deviation near the sanitary and precipitation drainage collectors. Utility installation seepage influence was differentiated by the increase in groundwater electrical conductivity measured at the pumping wells. Results show that not only distance between the wells, as the main parameter that affects the system, but also industrial utility lines can have an influence on thermal field breakthrough.

## 1. Introduction

Geothermal energy is a renewable and sustainable energy source, 50 thousand times larger than all oil and gas resources in the world [1]. As a clean, reliable, and abundant source of energy, it imposes itself as a solution to the problem of global warming, pollution, and rising prices of fossil fuels [2]. In nature, heat is transferred from a higher temperature to a lower temperature. The system that transfers energy in the opposite direction is called a heat pump [3]. The groundwater heat pump (GWHP), a type of ground source heat pump (GSHP), uses heat energy stored in the aquifer to heat and cool buildings. The GWHP consists of aquifer thermal energy storage (ATES), a heat pump unit, and a terminal air conditioning system. The ATES consists of one or more pairs of groundwater wells [4]. Compared to closed-loop systems, the GWHP can achieve a higher heat exchange rate (HEX). The reason for this is, unlike the closed-loop system [5], the temperature of the water circulating through the GWHP system is close to the mean groundwater temperature. The use of energy stored in the ground can result in savings of 80% on cooling and 30% on heating [6]. Despite its advantages, in the process of designing a GWHP system, it is necessary to consider its disadvantageous aspects, such as potential ground subsidence, clogging of the return well, and groundwater contamination. Additionally, in the long term, thermal breakthrough [7] can have a negative effect on the functionality and efficiency of the system.

**Figure 1:**
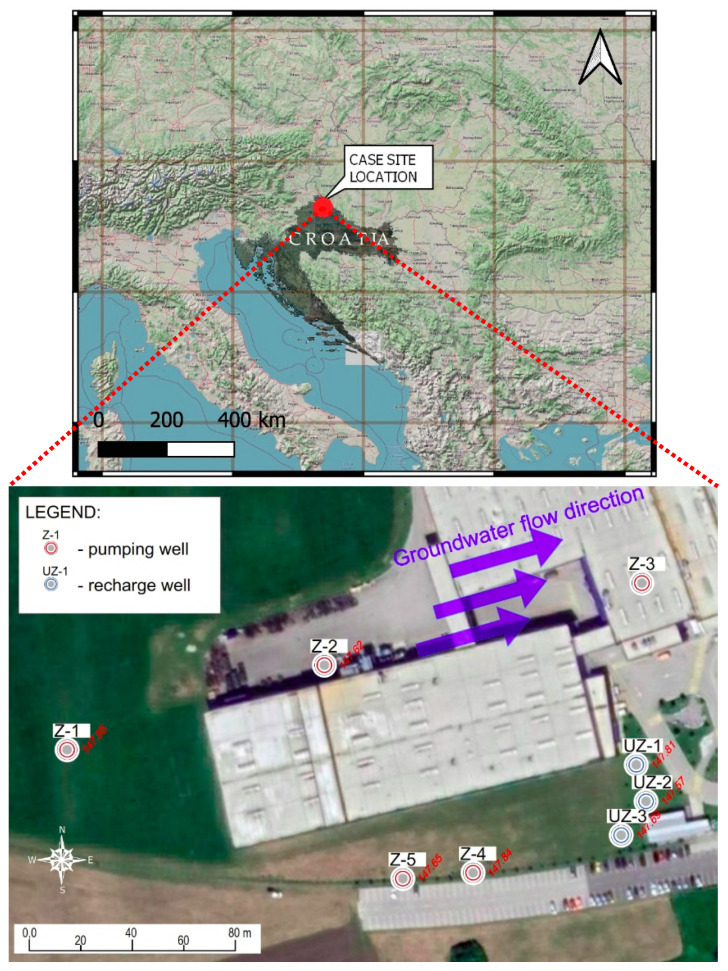
Case site location with groundwater flow direction and positions of wells.

This paper presents an example of a GWHP system constructed specifically for the purpose of heating and cooling a production hall. The production hall from this case study is in the Međimurje region, NW Croatia (Figure 1). The site is located on the edge of the Pannonian Basin, in the Varaždin depression [8]. The Varaždin depression occupies the Drava River plain and is separated from the hill areas in the north and south by faults. The aquifer system is composed of gravel and sand of the Quaternary age, deposited during the Middle and Upper Pleistocene and Holocene as the result of the accumulation process of the Drava River. In the deepest part of the depression, the thickness of the sediment is greater than one hundred meters [9]. The geological formations described represent favourable conditions for the construction of a GWHP system. The novelty of this study lies in the implementation of theoretical background of temperature breakthrough occurrence of a real case.

**Figure 2:**
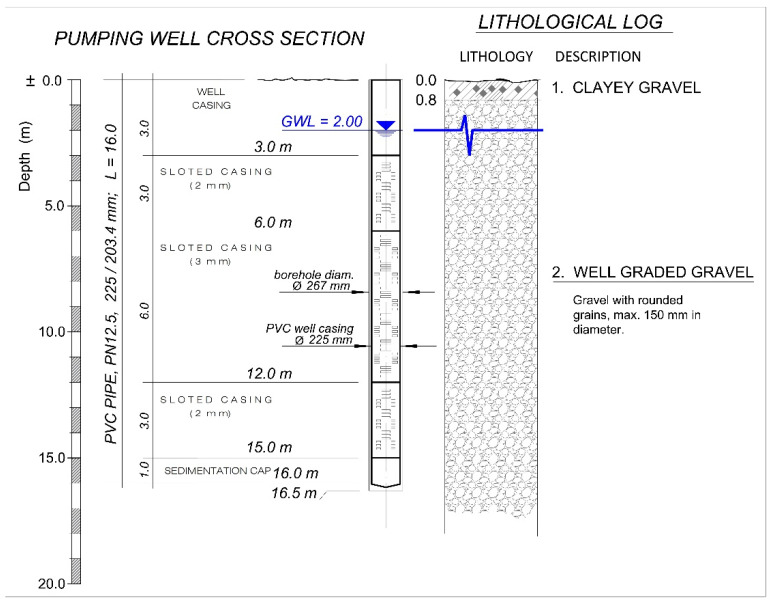
Lithological log and technical cross-section of the discharging and recharging well.

During previous investigations at the same location, exploratory borehole drilling was carried out to determine the detailed geological lithology. During drilling operations, core samples of the geological formations were collected and described lithologically. Laboratory analyses on the core samples were also conducted. An unconfined aquifer thickness is greater than 35 m. It was also determined that the upper part of the aquifer, up to a depth of 14 m b.g.s., is composed of well-graded gravel (i.e., poorly sorted gravel); whereas, the deeper layers consist of silty and clayey gravel. The covering layer of the aquifer is very thin, with a thickness of only 0.8 m. The direction of groundwater flow is W–E (Figure 1). The hydraulic conductivity of the upper part of aquifer material was 1000 m/day, as determined from a grain size distribution analysis of a sample. Figure 2 shows a lithological logging and technical cross-section of the discharging and recharging wells. PVC casing Ø 225 mm were installed in the wells.

Recent research has shown that during pumping wells’ exploitation, a significant influence of the recharging well, sanitary influence, and rain drainage can be registered. This results in the increase in the temperature from the pumping or discharging well, i.e., a thermal breakthrough was noticed. Temperature increment in the pumping well resulted in an overall decrease in the efficiency of the GWHP system. A detailed analysis of these claims is given in several diagrams, figures, and explanations.

## 2. Materials and Methods

### 2.1. Unconfined Aquifers and Aquifer Parameters

**Figure 3:**
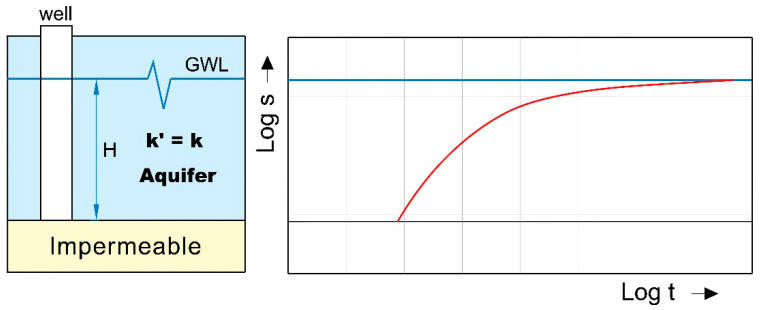
Time-drawdown curve in an unconfined aquifer.

Where groundwater is in direct contact with the atmosphere through the open pore spaces of the overlying soil or rock, the aquifer is said to be unconfined. An unconfined aquifer is an aquifer in which the water table forms the upper boundary. The upper groundwater surface level (GWL) in an unconfined aquifer is called the water table. The depth of the water table varies according to factors such as the topography, geology, season and tidal effects, and the quantities of water being pumped from the aquifer [10]. As unconfined aquifers usually have a high storage coefficient, it can take a very long time before the decline stabilizes. Unconfined aquifers are generally recharged by rain or stream water infiltrating directly through the overlying soil. Typical unconfined aquifers include coastal sands and alluvial deposits in river valleys [11]. An unconfined aquifer is a permeable layer on an impermeable substrate and partially saturated with water (Figure 3).

The fundamental property of an aquifer is to transmit, store, and yield water. An aquifer’s ability to do so is described through several parameters, such as hydraulic conductivity, transmissivity, and the storage coefficient. The hydraulic conductivity (*k*) indicates the aquifer’s ability to conduct water under a hydraulic gradient. It is a combined property of the porous medium and the fluid flowing through it [12] and depends on a variety of physical factors, including the porosity, particle size distribution, shape, and arrangement of particles, water density, temperature, and viscosity [11]. Transmissivity (*T*) is defined as the rate at which water can pass through the full thickness of the aquifer. Simply stated, it is the hydraulic conductivity multiplied by the thickness of the aquifer:

(1) T=k×b

where *k* is the hydraulic conductivity (m/day), *b* is the thickness of a confined aquifer, or saturated thickness of an unconfined aquifer (m).

The storage term for unconfined aquifers is known as a specific yield S_y_. It is defined as the volume of water that an unconfined aquifer releases from storage per unit surface area of the aquifer per unit decline of the water table. The specific yield of unconfined aquifers is much higher than the storability of confined aquifers. The usual range of S_y_ is 0.01–0.30. The higher values reflect the fact that a release from storage in unconfined aquifers represent actual dewatering of the soil pores, whereas a release from storage in confined aquifers represents only secondary effects of water expansion and aquifer material compaction [13]. The most reliable method for estimating aquifer parameters is by conducting pumping tests of the wells. Based on the observations of water drawdown levels near the wells, an integrated K value over a sizable aquifer section can be obtained. Unlike testing on a sample, pumping tests are conducted on an undisturbed aquifer in its natural hydraulic environment. The reliability of the determinations, made through the results of the pumping test, is far superior to laboratory methods [11]. Another advantage of the pumping test is the ability to determine the drawdown value as well as the extent/radius of the drawdown for the purpose of assessing the consequential subsidence of the surrounding terrain.

### 2.2. Pumping Tests

The purpose of a pumping test is to pump water from a well whilst monitoring the discharge quantities and water level drawdown in the same well, or better, in the neighbouring observation well or piezometer. By observing dynamic water levels on a remote observation well at a known distance from the pumping well, measurements can be inserted into an appropriate well-flow equation and calculate the hydraulic characteristics of the aquifer [14]. The pumping test of the discharging well is conducted at the test site. Two types of testing procedures are applied: a constant test and a step-drawdown test. The water level and discharge rate are recorded. The aquifer parameters are calculated by the well test analysis software, AquiferTest [15], which is a graphical interface for analysing test data from pumping tests, slug tests, and Lugeon tests with seamless integration with pumping test instrumentation. The basis of the test pumping is observing the drawdown of water in the well, i.e., on one or more control piezometers of various distances (r) from the well. Water lowering can be studied by constant step test or recovery test. The total drawdown (*s_t_*) in a pumping well can be expressed in the form of the following equation [16,17]:

(2) $$s_{t}=s_{a}+s_{w}=BQ+CQ^{2}$$

where:

*s_t_*—total drawdown in a pumping well (m);
*s_a_* = *BQ*—part of drawdown due to Aquifer losses, as laminar term;
*s_w_* = *CQ*^2^—part of drawdown due to well losses, as turbulent term;
*B*—factor related to the hydraulic characteristics of the aquifer (s/m^2^);
*C*—factor related to the characteristics of the well (s^2^/m^5^);
*Q*—pumping rate (m^3^/s).

**Figure 4:**
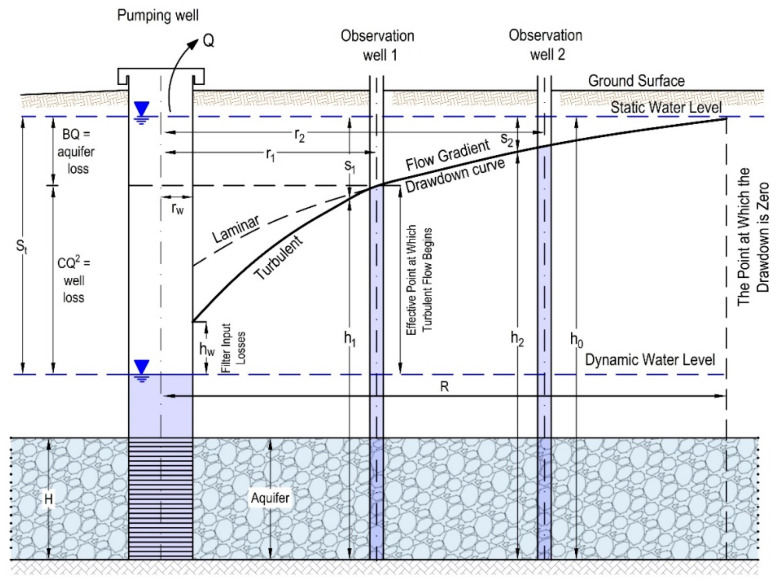
Drawdown in the pumping well.

Component BQ represents the loss in the aquifer in which the drawdown is caused by the resistance of the laminar flow in the aquifer, and component CQ^2^ represents the loss in the well caused by turbulent water flow in the filter part of the well structure and part of the aquifer (Figure 4). The drawdown values are required to calculate the layer resistance parameters (B) and well resistance (C). Drawdown parameters are calculated for different pumping volumes. Knowing parameters B and C, the drawdown in the well can be calculated for any amount of pumping. Since parameter C is not dependent on the pumping duration time, the well resistance reduction component is used to determine the total well drawdown [18].

The step-drawdown test was developed to assess the well performance and well losses due to turbulent flow. The well is pumped at several pumping rates, and drawdown for each step is recorded with time, each step lasting from 1 to 2 h. The step-drawdown test is used to determine the optimum pumping rate. The step-drawdown test can be used to determine transmissivity and total drawdown from each step [19].

The hydraulic conductivity (coefficient of permeability) *k* (m/s) can be calculated from the data on the drawdown of the water level in the piezometers during the pumping test and based on the flow measurement, according to the following expression (the markings are according to Figure 4):

(3) $$k=\frac{Q}{2\pi }\times \frac{ln\frac{r_{2}}{r_{1}}}{h_{w}\left(s_{1}-s_{2}\right)}$$

Good design of the filter part of the well structure can reduce losses to a greater extent, but it can never eliminate them. The relationship between the nonlinear well resistance parameter (C) and the well condition is given according to Walton [20] in Table 1.

By dividing Equation (2) by the pumping rate (*Q*), Equation (4) is defined. Another parameter can be computed from a step-drawdown test, according to Equation (5). In Equation (5), *L_p_* is the ratio of laminar head losses to the total head losses. This parameter can also be considered, as well as efficiency.

(4) s/Q=B+CQ

(5) $$L_{p}=\left(BQ/\left(BQ+CQ^{2}\right)\right)\times 100$$

Determining the optimum pumping rate is based mainly on the well losses and well efficiency. The procedure consists of the following steps:

1. Draw for up to more different *Q*, find s_T_ based on the well equation *s_t_* = *BQ* + *CQ*^2^.
2. For the same pumping rates, find theoretical (*s*) through the equation: *s* = (q/2πk) ln(R/r_w_), Figure 4.
3. Calculate well efficiency for all pumping rates.
4. Display the graph between efficiencies and pumping rates, and choose a *Q* value that corresponds to more than 65% efficiency or more.

This parameter can also be considered, as well as efficiency. Determining the optimum pumping rate is based mainly on the well losses and well efficiency. A well efficiency of 65% or more is usually acceptable. Step-drawdown tests are tests at different pumping rates (*Q*) designed to determine well efficiency, *L_p_*, normally pumping at each successively greater rate *Q*_1_ < *Q*_2_ < *Q*_3_ < *Q*_4_ < *Q*_5_, which takes place for 1–2 h (Δt) and takes 3 to 5 steps. The entire test usually takes place in one day.

**Figure 5:**
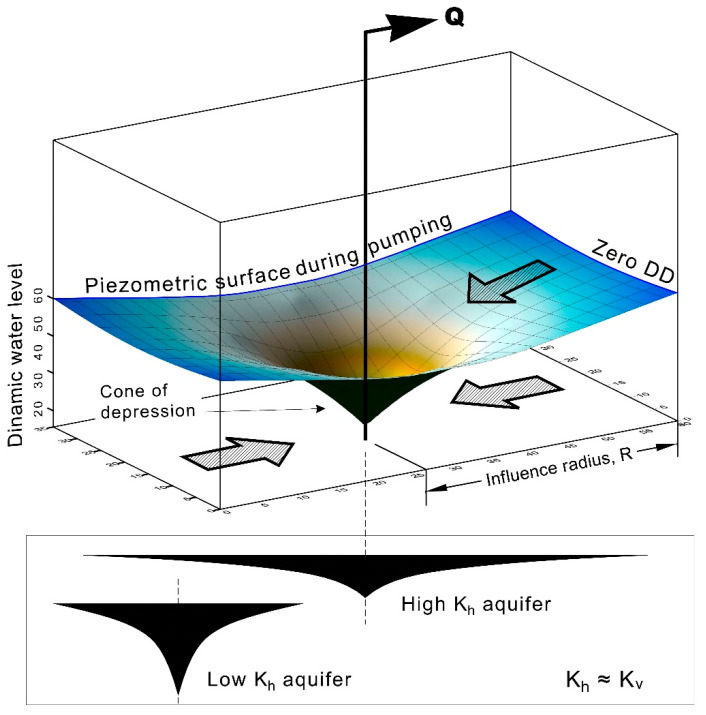
Diagram of a cone of depression and influence radius for pumping wells.

Head gradient decreases away from the well, and the pattern resembles an inverted cone called the cone of depression. A zone of low pressure is created centred on the pumping well (Figure 5). The cone expands over time until the inflows (from various boundaries) match with the discharging well. If the pumping lasts long enough, the depression cone reaches a steady state and no longer expands. When this is achieved, the amount of water that passes through the ring around the well and the depression cone is equal to the amount of water pumped. At this point, a state of equilibrium is reached. Many empirical formulas are provided, giving an approximation for the radius of influence. They are based on: mean grain diameter (d50), hydraulic conductivity, eater well discharge, and drawdown at the pumping well [21]. The following is only an overview:

(6) $$R=\sqrt{2.25Tt/S}$$

for confined aquifers after a short period of pumping [22].

(7) $$R=\sqrt{1.9kht/n}$$

for unconfined aquifers [23].

(8) $$R=3000\sqrt{k}$$

Sichardt formula, for unconfined aquifers [24]. Where:

*R*—influence radius (m); *T*—transmissivity (m/s); *t*—time (s); *S*—storage.
*h*—height of the water table above substratum (m); *n*—effective porosity.
*k*—hydraulic conductivity (m/s); *s*—drawdown in the borehole (m).

### 2.3. Velocity of Groundwater Flow Rate (Darcy’s Law)

**Figure 6:**
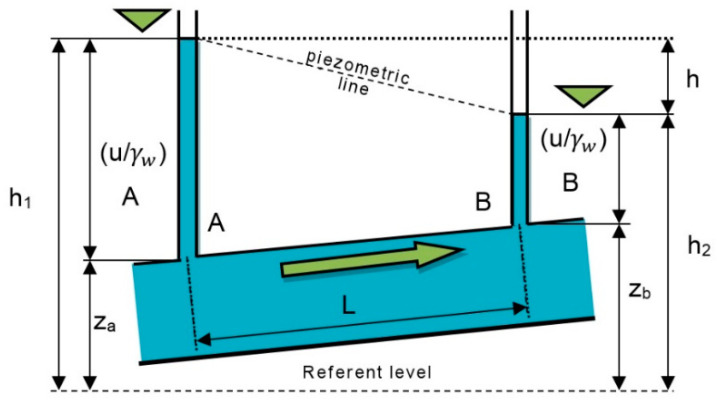
Darcy’s law—flow in porous medium.

French engineer Henry Darcy found that water flow through the soil is analogous to water flow in pipes. He performed a series of tests on a vertical pipe that was filled with sand. Through these experiments, he found that the flow rate through a column of saturated sand is proportional to the difference in hydraulic potential at the ends of the column and inversely proportional to the length of the column (Figure 6). It shows that the volumetric flow rate is a function of the flow area, elevation, fluid pressure, and a proportionality constant [25].

This realization, called Darcy’s law, is expressed through a basic equation that describes groundwater flow. This law can be mathematically expressed as:

(9) $$v=\frac{Q}{A}=k\times i\gg Q=k\times i\times A$$

where is:

*v*—Darcy velocity of flow (m/s);
*Q*—volume of water passing through the porous medium per unit time (m^3^/s);
*A*—cross-sectional area of the porous media (m^2^);
*k*—coefficient of permeability or coefficient of hydraulic conductivity (m/s);
*i*—hydraulic gradient, i = (h_1_ − h_2_)/L (−);
*L*—distance between piezometers (m).

Flux obtained from Equation (9) is the ideal velocity of groundwater. It assumes that water molecules can flow in a straight line through the subsurface. The actual groundwater flow (*v_x_*) is obtained when the value of the effective porosity (*n_e_*) is entered in the above expression, so we obtain:

(10) $$v_{x}=v/n_{e}$$

Effective porosity (*n_e_*) is generally defined for solute transport as that portion of the soil or rock through which chemicals move, or that portion of the media that contributes to flow [26,27]. Porosity is defined as the proportion of voids in the porous medium’s overall volume. Effective porosity can be obtained from field-scale well-tracer tests, in which a tracer is injected into a well and is pumped back from either the same injection well or from another well. For example, Hall et al. (1991) [28] proposed estimating effective porosity in a homogeneous confined aquifer dominated by steady-state horizontal advective transport with a constant hydraulic gradient. They use Darcy’s equation, with an added effective-porosity term from Equation (10).

**Figure 7:**
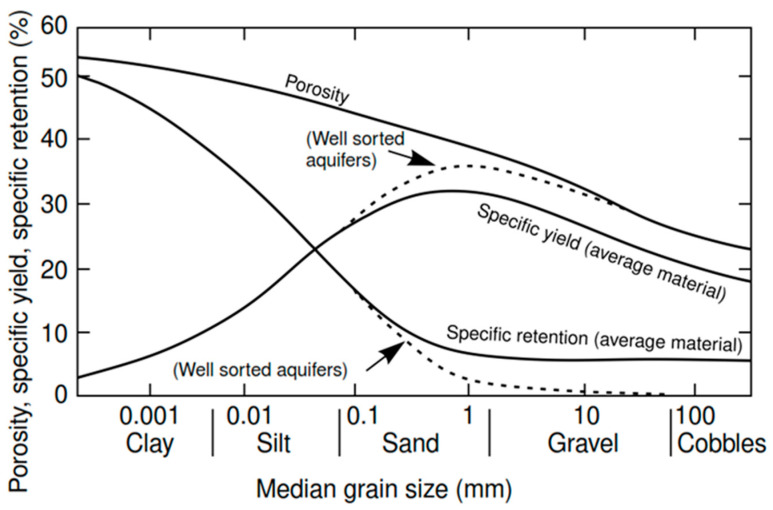
Relationship between median grain size and water-storage properties of alluvium.

This groundwater velocity accounts for the tortuosity of flow paths by including effective porosity (*n_e_*) in the calculation. Darcy’s law is used extensively in groundwater studies. It can help answer important questions such as the direction a pollution plume moves in an aquifer and how fast it travels. Effective porosity as required in groundwater transport models can be determined by laboratory and field techniques. Excellent discussions of effective porosity in transport processes are in [29,30]. To calculate Darcy’s and actual velocities, the effective porosity (*n_e_* = 0.30) was taken from Figure 7, since the aquifer was constructed of well-graded gravel [31].

Hence, it was concluded that aquifer can provide a significant groundwater yield per well. The estimate of the groundwater yield of the well is based on a maximum acceptable drawdown in the well. Considering a fully penetrating well, a commonly agreed drawdown limit is one-third of the saturated aquifer thickness of an unconfined aquifer [17]. The pumping test results showed a very small drawdown in the well.

The input parameters for determining the maximum capacity for the laminar flow of wells are primarily a function of the maximum velocity of the incoming water, which for the laminar flow is 0.030 m/s [17]. The maximum capacity per meter ($q/m^{\prime }$) for a given laminar water flow condition is calculated according to the following expression:

(11) $$q/m^{\prime }=d\times \pi \times v\times f$$

The maximum designed water supply capacity in the well for the laminar flow of water inlet into the filter part of the pipe is calculated according to:

(12) Q=d×π×v×f×L

**Figure 8:**
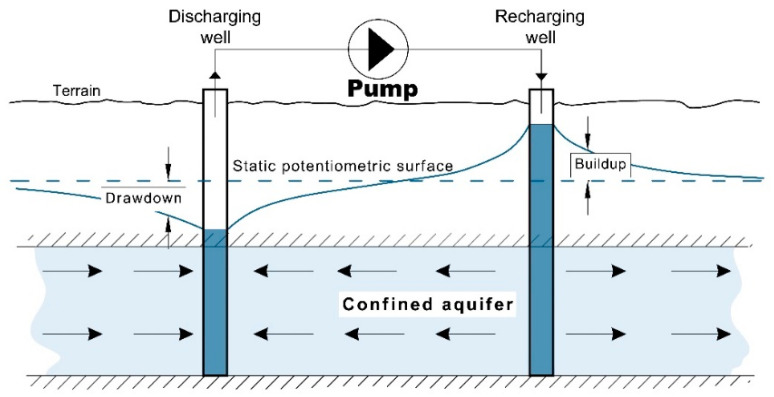
Wells with discharging/recharging quantities and drawdown/build-up from GWL.

At any point in an aquifer affected by both a discharging well and a recharging well, the change in water level is equal to the difference between the drawdown and the build-up. If the rates of discharge and recharge are the same and if the wells are operated on the same schedule, the drawdown and the build-up will cancel midway between the wells, and the water level at that point will remain unchanged from the static level, see Figure 8 [16].

### 2.4. Sensors Used for the Measurements

**Figure 9:**
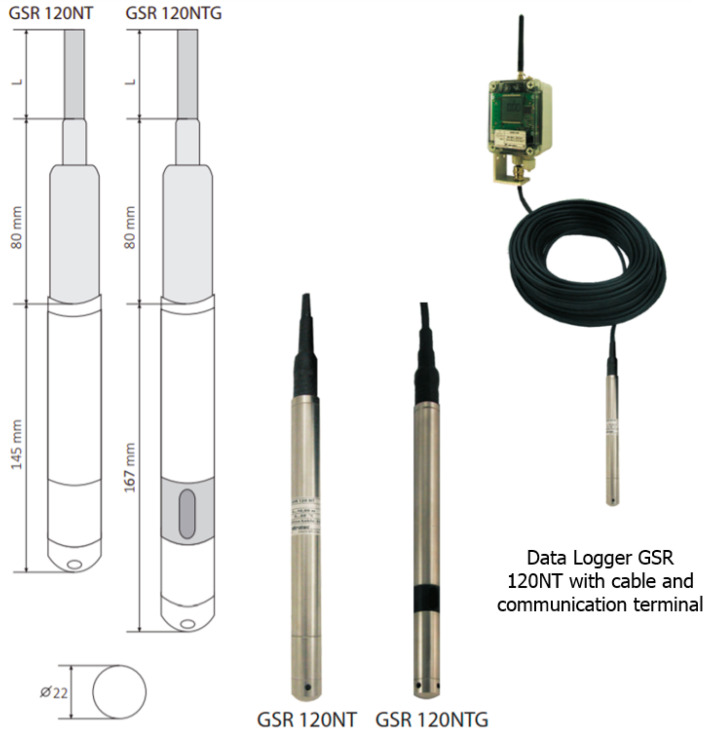
Sensors used in the measurement—GSR 120 NT and 120 NTG.

The sensors used in the measurements are shown in Figure 9. The manufacturer is Eltratecfrom Slovenia. The characteristics of the sensors, their use, and technical specifications are given in Figure 9.

Sensor characteristics:

− Level, temperature, and conductivity measurements;
− Compact submersible stainless steel housing;
− Battery or grid powered;
− Communication connection RS 485;
− Data transfer and parameter settings can be done with a computer (wired connection) or over a GSM modem (wireless connection);
Easy assembly.

Sensors monitor water (control of water levels in wells, boreholes, and piezometers), temperature, and conductivity of surface, groundwater, thermal, and industrial waters. The probe contains a microcontroller that stores the measured data from the sensor in certain time intervals in memory and, if necessary, communicates with a PC, laptop, or GSM/GPRS modem. The data logger can operate in three ways: locally (an independent data logger that can be connected to a PC on the build-in site; it can collect data and enter settings), network (up to 16 data loggers can be connected to an RS485 network so we can communicate with all the loggers from one site), or wireless (a GSM module is connected to the communication junction, which also houses batteries and an antenna, which enables a daily time set transfer of archived data to a selected remote location with a GSM signal).

### 2.5. Short Description of the Operation of the Cooling Station

**Figure 10:**
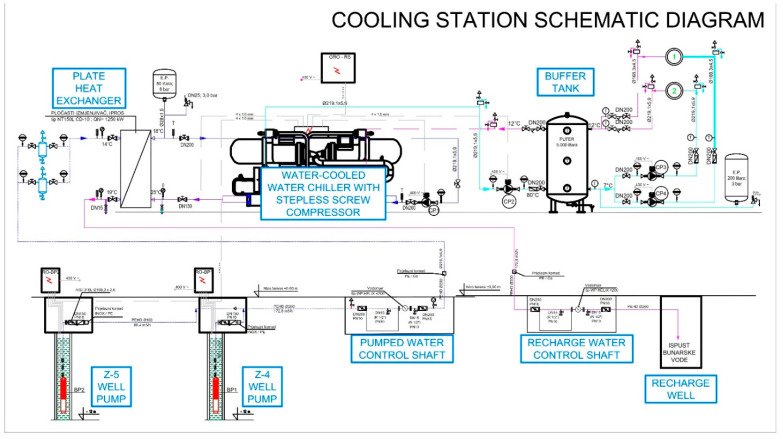
Cooling station schematic diagram for discharging wells Z-4 and Z-5.

From the schematic display of the cooling station and the way of using well water with the installed cooling system from Figure 10, the primary and secondary circuits of the cooler can be seen. The primary circuit of the cooler consists of pumping wells Z-4 and Z-5 (Figure 1), which supply the plate heat exchanger in front of the refrigeration unit. The plate heat exchanger is sized to achieve the required output of 1250 kW at a temperature difference of 2 °C (primary/secondary). 

The unfavourable influence of the environment and the proximity of pumping wells were considered during sizing. Pumps in Z-4 and Z-5 (Figure 10) are operated by the chiller unit and run depending on the temperature at the inlet to the cooler. On the primary side of the cooler, the working substance is heated (ecological Freon) with well water. If there is no consumption in the Buffer tank, cooling energy accumulates, and the system goes into standby mode. Depending on the temperatures in the production hall, the temperature in the recharging wells rises or falls.

## 3. Results

### 3.1. Results of Pumping Tests

**Figure 11:**
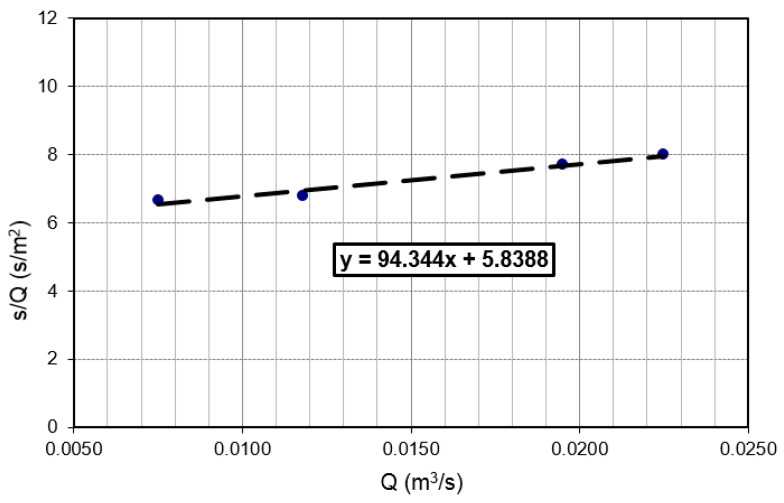
Values for B and C in the step test can be determined from a graph.

The factor related to the characteristics of the well C = 94.34 s^2^/m^5^ was obtained from Figure 11. When converted, the C equals to 0.03 min^2^/m^5^. According to Table 1, the pumping well Z-1 is well designed as the value for the well resistance parameter is C < 0.5. The depth of the discharging and the recharging wells was determined from the borehole log of an exploratory borehole; the installation depth is equal to L = 16.0 m. The step-drawdown test, through three steps, was carried out, together with a constant test, for approximately 10 h. Steady-state or equilibrium conditions occurred soon after the pumping test commenced. A maximum drawdown of only 0.12 m was observed in the Z-1 at a constant discharge rate of 26 L/s.

**Figure 12:**
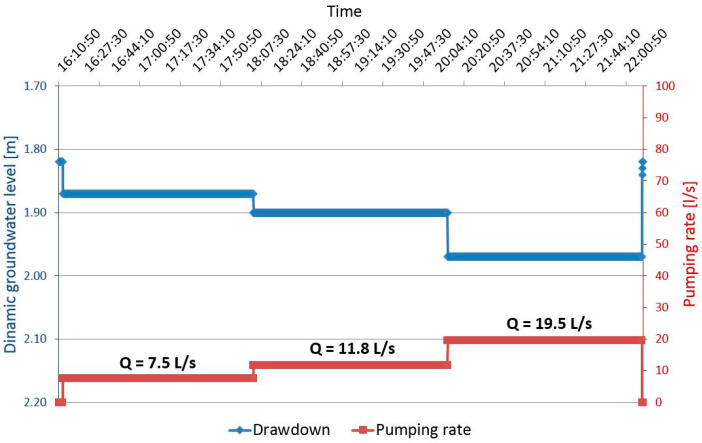
Drawdown in the pumping well Z-1 for step test.

**Figure 13:**
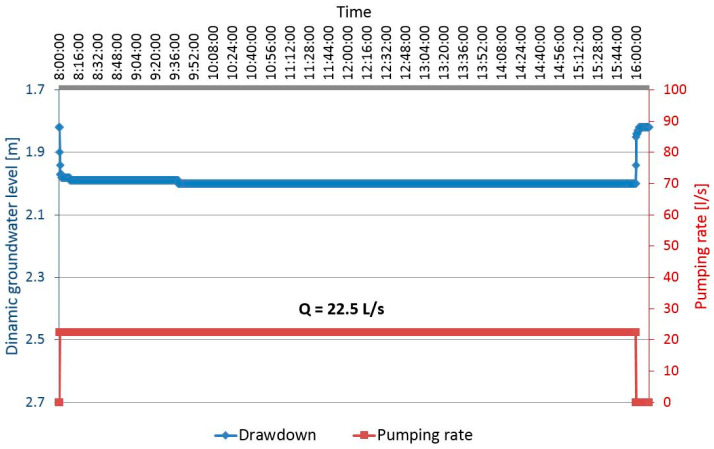
Drawdown in the pumping well Z-1 for constant test.

**Figure 14:**
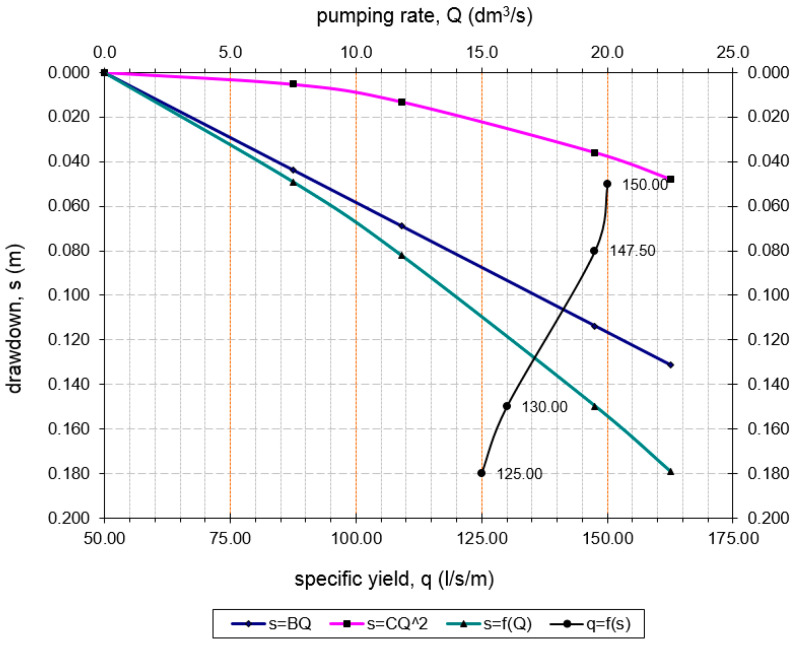
Losses in the pumping well Z-1 and the aquifer.

Figure 12 shows the drawdown for the discharging well Z-1 during the step test and Figure 13 the drawdown in well Z-1 at constant test. The drawdown in the pumping well shows minimal fluctuations in the dynamic water level during constant pumping with a uniform amount of Q = 22.5 L/s for 8 h. The equilibrium state of the pumped amount and recharge from the aquifer is achieved very quickly (Figure 13). Figure 14 shows the losses in the pumping well Z-1 and the aquifer.

**Figure 15:**
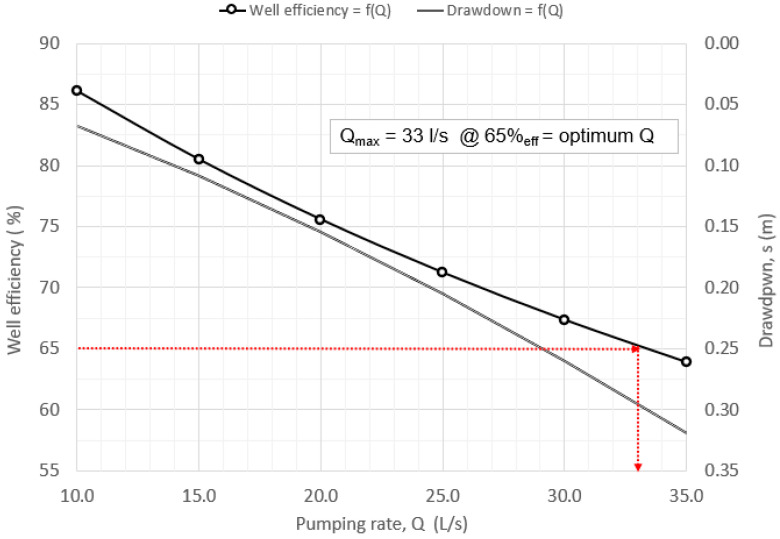
Determining the optimum pumping rate.

Equation (4) is a linear equation in s/Q and Q. If s/Q = f(Q), the resultant graph is a straight line with slope C = Δ(s/Q)/ΔQ. = 94.34 s^2^/m^5^ and intercept B = 5.83 s/m^2^ (Figure 11). The total drawdown expression: *s_t_* = 5.83·Q + 94.34·CQ^2^. In this case, for the pumping rate of Q = 26 l/s according to Equations (2), (4) and (5), well efficiency is *L_p_* = 70.38%. From Figure 15, the maximum acceptable pumping quantity Q_max_ = 33 l/s for well efficiency of 65% is obtained. The corresponding drawdown for this amount of pumping and the geometry of the well is only 0.30 m. A pumping rate that corresponds to more than 65% efficiency should be acceptable.

### 3.2. Hydrogeological Aquifer Parameters

The obtained value of the actual velocity according to Equation (10) is *v_x_* = 2.34 m/day (Table 2). Based on the absolute elevations of the groundwater level (GWL) in the wells, the direction of groundwater movement is from west to east. Hydraulic conductivity k (m/day) is 1000, Darcy’s velocity v (m/day) is 0.70, and actual velocity *v_x_* (m/day) is 2.34.

**Figure 16:**
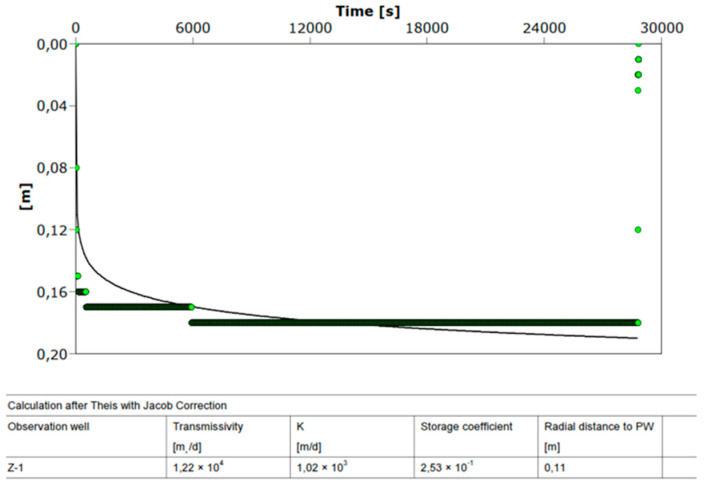
Aquifer parameters obtained at well Z-1, constant test (4 April 2017).

**Figure 17:**
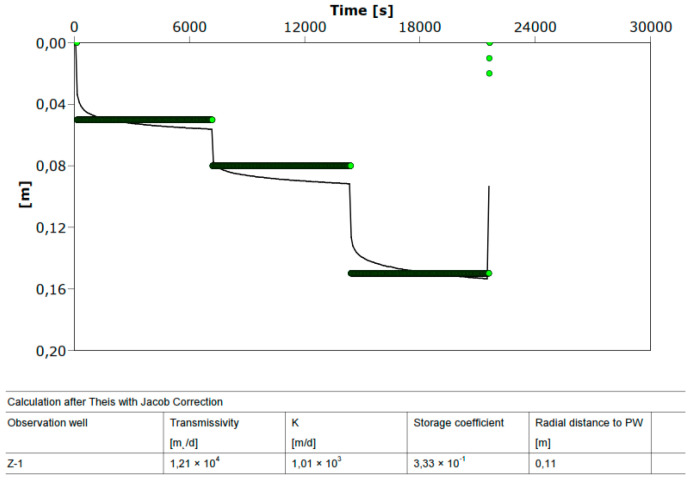
Aquifer parameters obtained at well Z-1, step test (4 April 2017).

**Figure 18:**
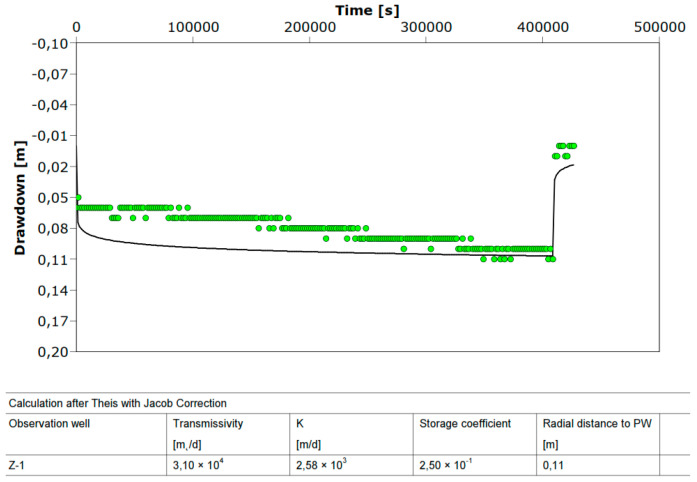
Aquifer parameters obtained at well Z-1, constant test (23 July 2021).

Hydrogeological parameters obtained by trial pumping at well Z-1 are given in Figure 16, Figure 17 and Figure 18. The test results (Figure 16 and Figure 17) listed in Table 3 show high hydraulic conductivity and transmissivity and the storage coefficient of the aquifer at the site. Figure 18 shows a constant test performed on the pumping well Z-1 from 23 to 28 July 2021. With the AquiferTest Pro program, the hydraulic conductivity K = 2580 m/day was obtained.

The parameters for calculating the maximum capacity per meter of filter for wells, according to Equation (11), are as follows:

*d* = 0.225 m—the diameter of the built-in filter;
*v* = 0.03 m/s—maximum velocity of water entry into the filter for laminar flow conditions;
*s* = 2.00 mm—the width of the strip opening (slot) of the filter;
*f* = 12.70 %—total perforated (slotted) part of the filter in percentages;
*L* = 12.00 m—the total length of the built-in filter;
$q/m^{\prime }$ = 2.69 l/s—specific capacity per meter length.

The maximum designed water supply capacity in the well Q = 32.28 l/s, for the laminar flow of water inlet into the filter part of the pipe, is calculated according to Equation (12). The calculated value deviates slightly from Figure 15, where the maximum acceptable amount of pumping Q = 33 l/s for well efficiency of 65% is obtained. Mentioned represents the lower value of acceptability of the maximum pumping capacity.

### 3.3. Results of Sensory Observation on Wells

**Figure 19:**
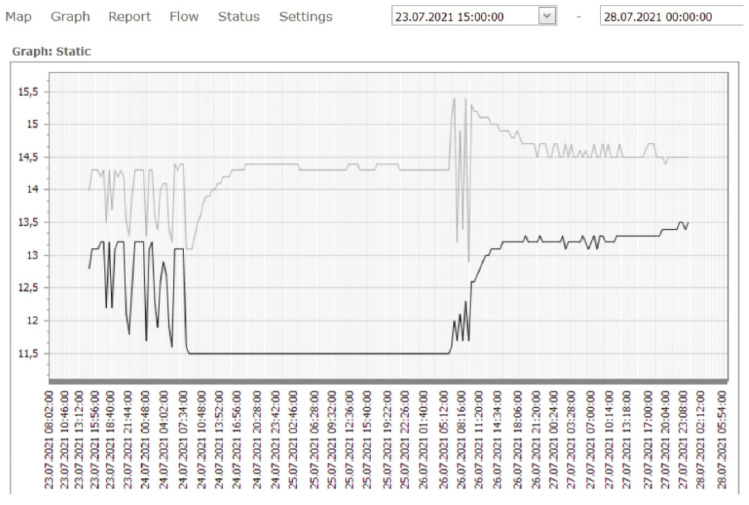

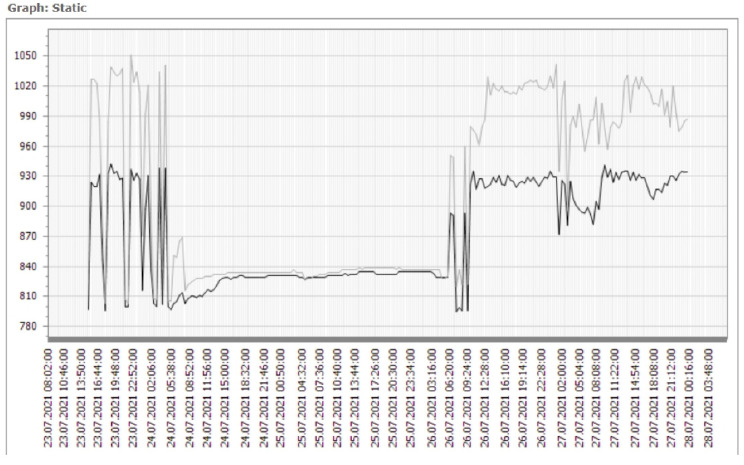
Original telemetry records for wells Z-4 and Z-5 (upper picture—water temperature, lower picture—electrical conductivity (µS/cm).

Figure 19 shows the data for water temperature and electrical conductivity. The sampling time was 30 min. The obtained data refer to the time interval from 23 July 2021 08:00 to 28 July 2021 23:00. In Figure 19, the black line refers to the pumping well Z-5 and the grey to Z-4. When the pumps in wells Z-4 and Z-5 do not work (steady state, 24 July 08:00 to 26 July 05:00), the water temperature in Z-5 is almost 3 °C lower than in well Z-4. The position of the wells is visible in Figure 1.

This indicates a significant temperature breakthrough in the Z-4 from the recharging wells and the sanitary and precipitation drainage collectors. It can be seen from Figure 19 that a significant temperature breakthrough occurs at well Z-5 (black line) when the pumps in Z-4 and Z-5 are switched on (after 26 July 2021 08:00). The sanitary and precipitation drainage collectors are located directly next to the wells (see Figure 10). A significant increase in electrical conductivity at the beginning of pump operation shows the impact of sanitary and precipitation drainage installations on the increase in wells Z-4 and Z-5, since their temperature is significantly higher than the groundwater temperature.

**Figure 20:**
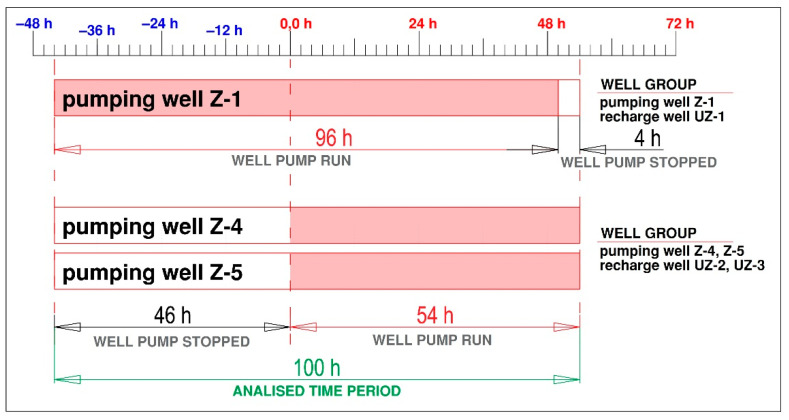
Pump operation schedule.

The observations were performed in phases and shown on the flow chart in Figure 20. Before the measurement, a precise height survey of the measured wells and static groundwater levels in the steady state was performed (see Table 2). A critical period is the 46-hour standstill of the system to achieve stationary conditions. It is necessary to control the pumps in wells Z-4 and Z-5 to achieve their simultaneous operation and the same amount of pumping, according to Figure 20.

## 4. Discussion

### 4.1. Analysis of Pumping Test Results

The hydraulic conductivity K = 2580 m/day obtained on the pumping well Z-1 from 23 to 28 July 2021 significantly higher than the one obtained on 4 April 2017 from K ≈ 1000 m/day. A higher hydraulic conductivity value also implies a lower drawdown of water in the well for the same amount of pumping. It can be seen from Figure 16 that the drawdown in the constant test with a pumping volume of 22.46 l/s was 18 cm (4 April 2017), and four years later, with a pumping amount of 25 l/s, the drawdown was only 11 cm (see Figure 18). The increase in hydraulic conductivity back four years by 2.5 times is due to a large amount of pumped water in Z-1, which resulted in the leaching of small particles (suffusion) and a decrease in the compaction of incoherent material (gravel). Then, due to a large amount of groundwater pumping, the soil changes from compacted to loose and, thus, increases permeability. The flow of groundwater causes suffusion through the pores of the coarse-grained skeleton, whereby smaller unbound particles move and are transported in the pores [32]. The removal of a smaller fraction of the aquifer causes a change in its permeability, which can become several times higher than the initial value (see Figure 16 and Figure 17). Due to a large amount of groundwater pumping, the soil gradually changes from compacted to loose. This is the reduced number of strokes of the standard penetration test (SPT) if they are performed near larger wells or water pumping stations.

### 4.2. Analysis Sensors Obtained Data

Figure 19 indicates a significant temperature breakthrough in the Z-4 from the recharging wells and the sanitary and precipitation drainage collectors. It can be seen from the figure that a significant temperature breakthrough occurs at well Z-5 (black line) when the pumps in Z-4 and Z-5 are switched on (after 26 July 2021 08:00, Figure 19 and Figure 20). The sanitary and precipitation drainage collectors are located directly next to the wells (Figure 10). A significant increase in electrical conductivity at the beginning of pump operation shows the impact of sanitary and precipitation drainage installations on the increase in wells Z-4 and Z-5, since their temperature is significantly higher than the groundwater temperature.

**Figure 21:**
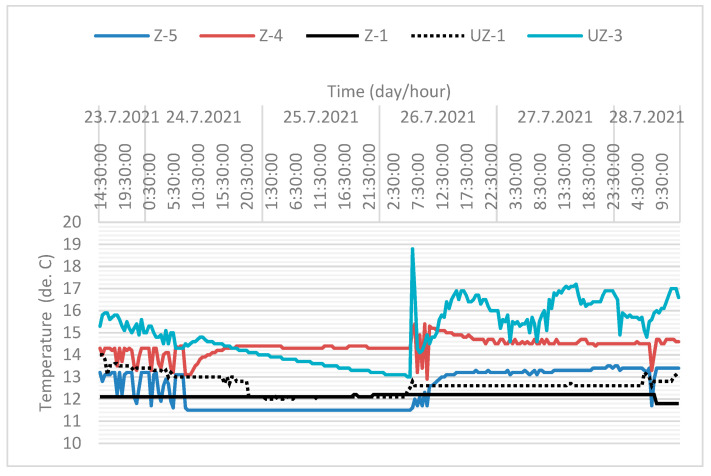
Groundwater temperatures during pumping in the observed wells.

Based on the analysis of the obtained values, it can be seen that when monitoring groundwater at steady-state intervals 24 July 2021 8.00 to 26 July 2021 6:00, Figure 21 (pumps do not work), we have differences in temperature at wells Z-4 and Z-5 up to 3.0 °C. Groundwater temperatures in the measured wells are shown in Figure 21.

In the wire processing plant, the exploitation well Z-1 has the function of cooling the produced wire. Its associated discharging well is UZ-1. The temperature varied slightly from the initial well temperature Z-1 (12 °C), as it did not have a cooling function during monitoring. The slight increase in temperature in well UZ-1 was influenced by the temperature from the nearby recharging wells UZ-2 and UZ-3, where the temperature rises due to the start working wells Z-4 and Z-5 (Figure 21).

**Figure 22:**
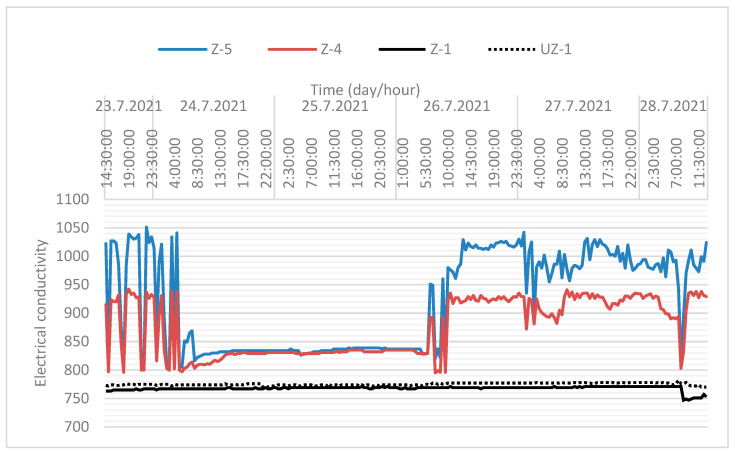
Display of electrical conductivity (µS/cm) in wells during pumping.

The electrical conductivity of groundwater was also measured in wells Z-5, Z-4, Z-1, and UZ-1, (Figure 22). The electrical conductivity of water is the ability to conduct electricity. It depends on the presence of ions, their total concentration, and the temperature of the measurement. The amount of solute in water significantly affects its conductivity. The unit of electrical conductivity is micro-Siemens per centimetre (µS/cm). The electrical conductivity values of drinking water range from approximately 200–800 µS/cm.

**Figure 23:**
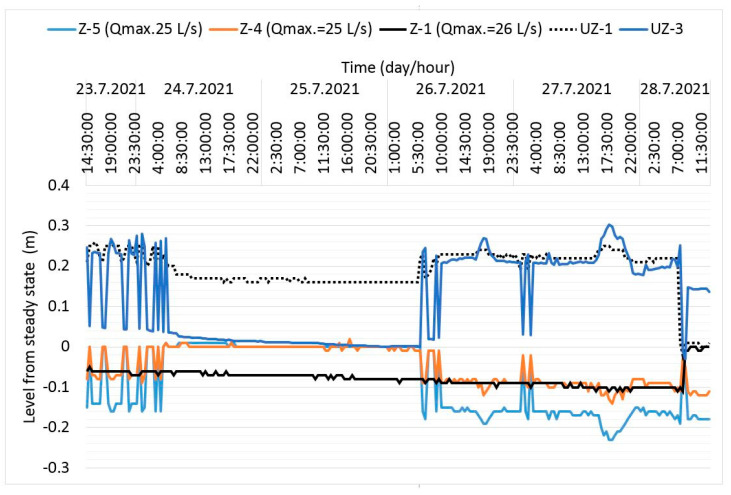
Display of groundwater drawdown/build-up during pumping in the observed wells.

Figure 23 shows that during pumping with the maximum pumping amount of Q = 25 l/s in well Z-5, there was a decrease of −0.23 cm (27 July 2021 18:30) from the initial steady state. For the same amount of pumping in Z-4, the drawdown was only −0.14 cm. The reason for this is the slightly higher specific yield of Z-4 micro-locations. In the case of well Z-1, which pumped the amount of groundwater of Q = 26 l/s, the drawdown was only −0.12 cm (28 July 2021 8:00, see Figure 23). The reason for that is explained in more detail in Section 2.3 and Section 3.2.

**Figure 24:**
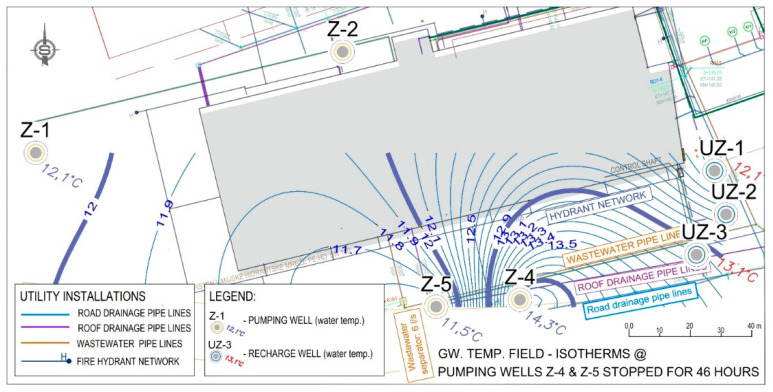
State of the thermal field (isotherms) in the aquifer, pumps Z-4 and Z-5 do not work.

**Figure 25:**
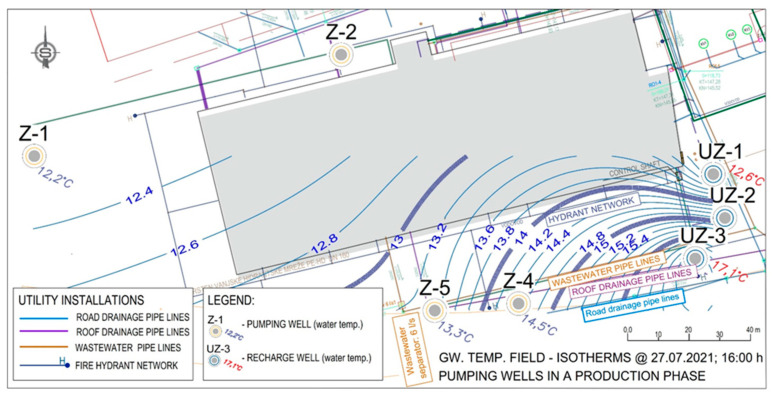
The state of the temperature distribution (thermal field) for the regular mode of operation on 27 July 2021 at 16:00.

**Figure 26:**
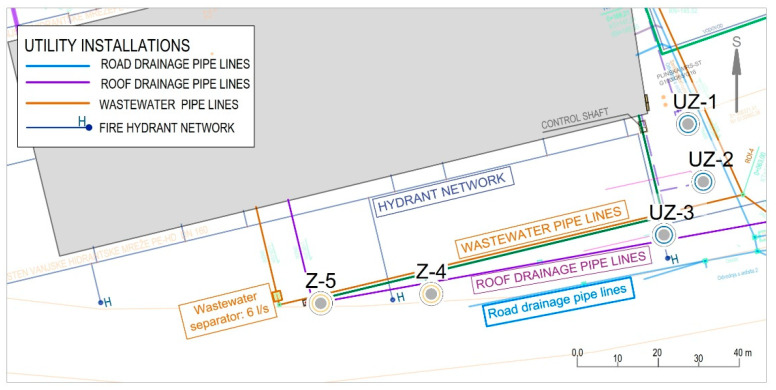
Overview of water supply installations and sanitary and precipitation drainage.

The data obtained by measuring the water temperature in the observed wells from Figure 22 were used to make maps of isotherms (Figure 24 and Figure 25). Figure 26 was used as the basis for Figure 24 and Figure 25. When researching the thermal field in the aquifer, water temperatures were observed inside the constructed wells that make up the LPT Prelog plant. Figure 24 shows the deviation of the thermal field when the pumps are at rest in the space between wells Z-4 and Z-5. The temperature difference between these wells is Δt = 2.8 °C (Figure 19). Such a steep thermal gradient is a consequence of local influences on groundwater temperature. As there are several infrastructure lines with liquids of higher temperature in the immediate vicinity, it can be assumed that the local influence is precisely the installation lines, i.e., in this case, the collectors of sanitary and precipitation drainage.

The isotherms in Figure 24 also clearly show how the thermal field was expected to form from the pumping wells to the recharging wells. The thermal field gradient records the expected distribution in the Z-1 well space.

During the operation of the cooling system, the affected groundwater at the pumping wells is returned with an elevated temperature to the aquifer through the discharging wells. The difference between the flow and return temperatures introduces changes in the thermal field of the aquifer. The magnitude of the change depends on the characteristics of the aquifer, the amount of water affected, the area of the affected area, etc.

**Figure 27:**
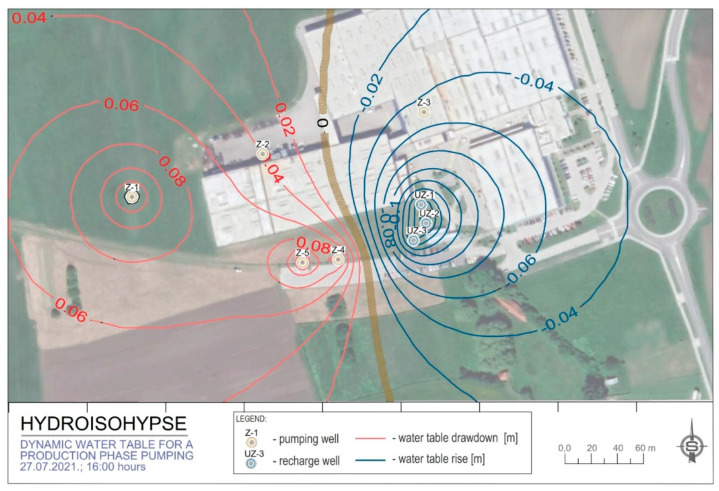
Hydroisohypses of relative drawdown/build-up of the water surface for the dynamic state of water pumping and discharging on 27 July 2021 at 16:00.

The measurements of the dynamic groundwater level (DGWL) from Figure 23 were used to make the map of the hydroisohypses (Figure 27). Figure 23 shows the dynamic levels of the water body in the regular regime of groundwater pumping of the production plant LPT, business zone Hrupine—Prelog. The state shown represents approximately the peak values of the pump quantities in cooling mode. Red hydroisohypses are shown with a positive sign and represent a decrease in the water level compared to the stationary level due to groundwater pumping. The blue lines have a negative sign and represent the build-up (camber) of the water face. The values along the isolines in the figure are expressed in units of the meter. The zero lines were formed in the north–south direction and connect the cones of the water face of different signs.

Table 4 gives the coordinates of all discharging and recharging wells. These data were also used to make the map of hydroisohypses, which are shown in Figure 27.

Most of the pumped groundwater is returned to the aquifer after the heat exchanger and is used as a cooling medium. Dynamic changes in groundwater levels have a limited impact and are partially extending beyond the ownership plots of the LPT Prelog plant. The spatial effects on the groundwater level due to discharging and recharging wells is shown in Figure 27.

A favourable characteristic of the groundwater project of the LPT Prelog plant is that the production is carried out in shifts, so the need for cooling is periodic and depends on the operating cycle. Thus, the system of groundwater use is occasionally shut down but also regulated relevant to needs, which is why the aquifer is quickly regenerated to a steady state.

## 5. Conclusions

A significant change in the thermal gradient was recorded towards the pumping wells Z-4 and Z-5. The increase in temperature is the previously mentioned sanitary and surface drainage collectors and the short distance between the recharge well U-3 and the pumping well Z-4. The increase in the electrical conductivity indicated the source of temperature breakthrough from the drainage collectors, as they also have a temperature higher than the groundwater.

Drawdowns in pumping wells and build-ups in recharging wells take on relatively small values, since the aquifer permeability is high. The measured coefficient of hydraulic conductivity exceeds the value of K > 1000 m/day. The obtained value of the actual groundwater flow velocity is *v_x_* = 2.34 m/s. Based on the absolute elevations of the groundwater level (GWL) in the wells, the direction of groundwater movement was defined from west to east.

Pumping well maximum acceptable pumping quantity was determined to be Q_max_ = 33 l/s for the required well efficiency of 65%. The corresponding drawdown for this amount of pumping is only 0.30 m. Additionally, the calculated allowable maximum pumping capacity for well filter laminar water inlet flow (0.03 m/s), according to Equation (12), is Q = 32.28 l/s. Both measured and calculated maximum pumping rate quantities are higher than the installed well pump capacity of Q = 25–26 l/s.

The hydroisohypses map obtained for the actual water pumping capacity (Q = 25–26 l/s) shows that the relative drawdown and build-up of groundwater levels outside the LPT Prelog plant perimeter are only a few centimetres. It will not affect the Prelog water pumping station, which is located at a distance of 1.00 km. From the stated, it can be concluded that in addition to the existing wells within the LPT factory, additional pumping volumes from new wells Z-4 and Z-5 up to 500,000 m^3^/year will not significantly impact the surrounding users.

From the diagram of the water temperature inside the wells, it is evident that at pumping, the temperature inside the well Z-4 is higher than Z-5 by approximately 1 °C. The same fact is not worrying because groundwater it is not used as “passive cooling”, and the designed system intake temperature is 14–15 °C. In addition, water from both wells is merged into a single system pipe inlet. It can also be seen that the temperature of the water discharged into the environment with the pumped water does not exceed the permitted 5 °C difference. Thus, it can be concluded that the system works correctly and has no impact on the environment. In the long term, thermal breakthrough cannot have a negative effect on the functionality and efficiency of the system.

However, if it is necessary to eliminate the temperature breakthrough on the pumping wells Z-4 and Z-5 and increase utilization in the heating and cooling system, it is proposed to move pumping wells to the location next to pumping well Z-1. The relocation would significantly improve pumped groundwater quality, as the new position of the wells is located away from utility installations.

## Figures and Tables

**Table 1:** Relationship between parameter C and well condition (Walton, 1970).

Well Loss C (min^2^/m^5^)	Well Conditions
<0.5	Good design and yield
0.5–1.0	Medium losses due to colmation
1.0–4.0	Seriously impaired due to colmation
>4.0	Very hard to regenerate to its original state

**Table 2:** Determination of Darcy’s and actual groundwater flow rate.

Well	Well Coordinates (HTRS96/TM)			GWL (Relatively)	GWL (Absolutely)	Well Distance (m)	ΔGWL (m)	i (-)	$\dot{x}$
	E	N	H						i (-)
Z-5	508,280.62	5,133,821.51	147.65	−2.130	145.515				
						27.30	0.0250	0.0009	0.0007
Z-4	508,307.86	5,133,823.74	147.84	−2.350	145.490				
						164.70	0.1010	0.0006	
Z-1	508,150.29	5,133,871.66	147.55	−1.960	145.591				
						221.10	0.1310	0.0006	
UZ-1	508,371.35	5,133,865.71	147.81	−2.350	145.460				
						127.20	0.0088	0.0007	
Z-2	508,250.17	5,133,904.50	147.62	−2.070	145.548				

Note: The coefficient of effective porosity in the calculation was taken n_ef_ = 0.30.

**Table 3:** Aquifer parameters obtained at well Z-1.

Test	*k* (m/Day)	*T* (m^2^/Day)	*S*
Constant	1020	12,200	0.253
Step	1010	12,100	0.330

**Table 4:** Position of wells with discharging/recharging quantities.

Well	Wells Coordinates (HTRS96/TM)		Q (l/s)	Drawdown/Build-Up (m)
	E	N		
Z-1	508,150.28	5,133,871.66	26	0.12
Z-2	508,250.17	5,133,904.49	1	0.04
Z-3	508,369.00	5,133,940.00	1	−0.05
Z-4	508,307.86	5,133,823.73	25	0.09
Z-5	508,280.62	5,133,821.51	25	0.12
UZ-1	508,371.35	5,133,865.71	−26	−0.20
UZ-2	508,375.23	5,133,851.64	−25	−0.20
UZ-3	508,365.56	5,133,838.46	−25	−0.18

## Data Availability

Data are available upon reasonable request towards corresponding author.

## References

[1] Garman D.K. (2004). Geothermal Technologies Program: Buried Treasure: The Evironmental, Economic and Employment Benefits of Geothermal Energy.

[2] Younis M., Bolisetti T., Ting D.S. (2010). Ground source heat pump systems: Current status. Int. J. Environ. Stud..

[3] Bundschuh J., Chen G. (2014). Sustainable Energy Solutions in Agriculture.

[4] Sommer W.T., Valstar J., Leusbrock I., Grotenhuis J.T.C., Rijnaarts H.H.M. (2015). Optimization and spatial pattern of large-scale aquifer thermal energy storage. Appl. Energy.

[5] Ooka R., Nam Y., Shiba Y., Tanifuji K., Okumura T., Miwa Y. (2011). Development of groundwater circulation heat pump system. HVAC&R Res..

[6] Schout G., Drijver B., Gutierrez-Neri M., Schotting R. (2014). Analysis of recovery efficiency in high-temperature aquifer thermal energy storage: A Rayleigh-based method. Hydrogeol. J..

[7] Ganguly S., Mohan Kumar M.S., Date A., Akbarzadeh A. (2017). Numerical investigation of temperature distribution and thermal performance while charging-discharging thermal energy in aquifer. Appl. Therm. Eng..

[8] Marković S., Mioč P. (1989). Basic Geological Map of SFRY 1:100000, Geology of the Nađ-Kaniža sheet.

[9] Urumović K., Hlevnjak B., Prelogović E., Mayer D. (1990). Hydrogeological Conditions of the Varaždin Aquifer. Geološki Vjesn..

[10] Confined and Unconfined Aquifers. https://dpipwe.tas.gov.au/water/groundwater/aquifers/confined-unconfined-aquifers.

[11] Todd D.K., Mays L.W. (2005). Groundwater Hydrology.

[12] Bear J. (1972). Dynamics of Fluids in Porous Media. Soil Sci..

[13] Freeze R.A., Cherry J.A. (1979). Groundwater.

[14] Kruseman G.P., de Ridder N.A., Ridder N.A., Verweij J.M. (1990). Improvement Analysis and Evaluation of Pumping Test Data.

[15] Hydrogeologic W. Aquifer Test. https://www.waterloohydrogeologic.com/products/aquifertest/.

[16] Heath R.C. (1983). Basic ground-water hydrology. Geological Survey Water-Supply Paper.

[17] Driscoll F.G. (1986). Groundwater and Wells.

[18] Jacob C.E. (1950). Flow of Groundwater in Engineering Hydraulic.

[19] Fetter C.W. (1994). Applied Hydrogeology.

[20] Walton W.C. (1971). Groundwater resource evaluation. J. Hydrol..

[21] Fileccia A. (2015). Some simple procedures for the calculation of the influence radius and well head protection areas (theoretical approach and a field case for a water table aquifer in an alluvial plain). Acque Sotter. Ital. J. Groundw..

[22] Copper H.H., Jacob C.E. (1946). A generalized graphical method for evaluating formation constants and summarizing well-field history. Eos. Trans. Am. Geophys. Union.

[23] Aravin V.I., Numerov S.N. (1953). Theory of motion of liquids and gases in undeformable porous media. Gostekhizdat Mosc..

[24] Cashman P.M., Preene M. (2001). Groundwater Lowering in Construction.

[25] Darcy H. (1856). Les Fontaines Publiques de la Ville de Dijon: Exposition et Application.

[26] Fetter C.W. (1993). Contaminant Hydrogeology.

[27] Domenico P.A., Schwartz F.W. (1991). Physical and Chemical Hydrogeology. Geol. Mag..

[28] Hall S., Luttrell S., Cronin W.E. (1991). Method for estimating effective porosity and ground-water velocity. Groundwater.

[29] Norton D. (1979). Transport Phenomena in Hydrothermal Systems: The redistribution of Chemical Components around Cooling Magmas. Bulletin de Minéralogie.

[30] Peyton G., Gibb J., LeFaivre M., Ritchey J. On the concept of effective porosity and its measurement in saturated fine-grained porous materials. Proceedings of the 2nd Canadian/American Conference of Hydrology.

[31] Stephens D.B., Hsu K.-C., Prieksat M.A., Ankeny M.D., Blandford N., Roth T.L., Kelsey J.A., Whitworth J.R. (1998). A comparison of estimated and calculated effective porosity. Hydrogeol. J..

[32] Muckenthaler P. (1989). Hydraulische Sicherheit von Staudämmen.

